# Behavioral and neurochemical alterations induced by long-term exposure to tobacco heating system emissions in rats

**DOI:** 10.3389/fphar.2025.1705059

**Published:** 2025-11-26

**Authors:** Rajeev Sood, Devesh Tewari, Musarrat Husain Warsi, Anoop Kumar, Shweta Kasna, J. P. Jaiswal, Pranay R. Sakya, Sonam Yoezer, Gaurav K. Jain

**Affiliations:** 1 Baba Farid University of Health Sciences, Faridkot, Punjab, India; 2 Department of Pharmacognosy and Phytochemistry, School of Pharmaceutical Sciences, Delhi Pharmaceutical Sciences and Research University, New Delhi, India; 3 Department of Pharmaceutics and Industrial Pharmacy, College of Pharmacy, Taif University, Taif, Saudi Arabia; 4 Department of Pharmacology, Delhi Pharmaceutical Sciences and Research University, New Delhi, India; 5 Centre for Advanced Formulation Technology, Department of Pharmaceutics, Delhi Pharmaceutical Sciences and Research University, New Delhi, India; 6 Norvic International Hospital, Kathmandu, Nepal; 7 Peoples Dental College Hospital, Kathmandu, Nepal; 8 Dechencholing Hospital, Thimphu, Bhutan

**Keywords:** nicotine, dopamine, mainstream emissions, cigarette, tobacco heating system

## Abstract

**Background:**

Tobacco heating systems (THSs) are increasingly being used as alternatives to conventional cigarettes (CIGs). In this study, we compared the effects of mainstream THS emissions and CIG smoke on the affective, locomotor, and cognitive behaviors in rats.

**Methods:**

Forty-five male Wistar rats were randomized into three groups (n = 15 each): control (normal air), CIG (mainstream smoke from a common cigarette brand), and THS (emissions from IQOS ILUMA and TEREA Smartcore Sticks™, Philip Morris International). Rats underwent whole-body exposure to emissions/smoke (two sticks/day, ∼4 min/stick, morning and evening) for 60 days. The toxicants were quantified in emissions/smoke. Post-exposure assessments included the actophotometer (locomotor activity), forced swim test (FST) (affective behavior), and novel object recognition test (NORT) (recognition memory). Blood was analyzed for hemoglobin (Hb), leukocyte count, serum nicotine, and serum cotinine. Brain dopamine levels and histopathological changes in the hippocampus and cerebral cortex were evaluated.

**Results:**

THS emissions exhibited significantly lower nicotine-free dry particulate matter (NFDPM), particulate matter, and toxicants (e.g., nitrogen/sulfur oxides, ammonia, hydrogen cyanide, and arsenic) than CIG smoke (p < 0.001). Behavioral analyses revealed that both exposure groups showed elevated locomotor activity and reduced immobility time in the FST compared to the controls, suggesting increased central arousal and reduced depressive-like behavior. The THS group demonstrated a higher discrimination index in the NORT, indicating relatively preserved recognition memory compared with that of the CIG group. Despite lower serum nicotine and brain dopamine levels, THS-exposed rats exhibited milder histopathological changes without apparent neurotoxicity, whereas CIG exposure induced marked hippocampal and cortical toxicity. CIG rats also showed elevated leukocyte counts and Hb relative to THS and control rats (p < 0.05).

**Conclusion:**

The observed behavioral effects likely reflect improved affective and locomotor regulation, with evidence of relatively preserved cognitive function in THS-exposed rats. These findings highlight the need for further mechanistic investigations to delineate the long-term neurobehavioral safety profile of THS exposure.

## 1 Introduction

Tobacco smoking remains a significant global public health issue and is responsible for over 8 million deaths annually (Ismail et al., 2024). Tobacco smoking generates a highly complex mixture of compounds, many of which have known toxic and carcinogenic effects (Soleimani et al., 2022). Nicotine is the primary addictive and active constituent of cigarette (CIG) smoke (Benowitz, 1992). In addition to being addictive, nicotine is well known to have serious systemic side effects. It adversely affects the cardiovascular (Zong et al., 2024), reproductive (Firouzabadi et al., 2025), cerebrovascular (Dennison Himmelfarb et al., 2025), and pulmonary systems (Allbright et al., 2024). Although nicotine use is associated with several health complications (Cao et al., 2024; Rose et al., 2010), increasing evidence suggests that nicotine has beneficial effects on memory and cognitive performance (Alhowail, 2021; Valentine and Sofuoglu, 2018). Swan et al. showed that acute administration of nicotine enhances attention, working and verbal memory, and executive function in adult nonsmokers (Swan and Lessov-Schlaggar, 2007). It was proposed that the performance-enhancement effect is facilitated by the activation of the cholinergic system and the subsequent effects on the release of dopamine (Swan and Lessov-Schlaggar, 2007). Nicotine has been shown to improve attention and cognitive function in smokers (Warburton, 1994). In another study, subcutaneous administration of nicotine was shown to improve attention-related task performance in Alzheimer’s disease (Jones et al., 1992). Alhowail concluded that nicotine has beneficial effects on memory and cognition (Alhowail, 2021). Despite the beneficial effects of nicotine on cognitive functions, CIG smoking is largely associated with memory and cognitive decline (Campos et al., 2016). The majority of research revealed that regular smokers score worse on neuropsychological tests than nonsmokers in terms of attention, cognitive function, memory, intellectual capacity, and executive functions (Campos et al., 2016; Conti et al., 2019; Durazzo et al., 2010). It is well known that CIG smoke, in addition to nicotine, contains numerous chemicals, many of which are toxic (Sood et al., 2024). Oxidants of nitrogen, sulfur dioxide, ammonia, hydrogen cyanide, acrolein, particulate matter, and heavy metals present in the smoke have been linked to neurotoxicity and cognitive decline (Campos et al., 2016). This might be a probable explanation for the association of nicotine, but not cigarette smoke, with the improvement in memory and cognition.

Recently, there has been growing interest toward alternative methods of nicotine delivery that could reduce the harmful health effects associated with traditional CIG smoking. One such innovative technology involves tobacco heating systems (THSs), with IQOS®, developed by Philip Morris International (Switzerland) as one of the most popular examples (Leigh et al., 2024). THSs are gaining popularity around the globe (Table 1), and their market size was valued at nearly $ 49 billion in 2024 and is projected to grow at a CAGR of 63.2% from 2025 to 2030 (Liu and Filippidis, 2024).

**TABLE 1:** Commercially available tobacco heating systems by manufacturers.

Manufacturer	Product name(s)	Heating mechanism	Tobacco stick brand
Philip Morris International (PMI)	IQOS ILUMA, IQOS ORIGINALS	Induction and resistive blade	TEREA, HEETS
British American Tobacco (BAT)	glo Hyper, glo Pro	Induction heating	Neo
Japan Tobacco International (JTI)	Ploom X, Ploom TECH+	Heating chamber	Mevius, Camel
KT&G (South Korea)	lil SOLID, lil HYBRID	Blade heating	Fiit
China Tobacco/Sixhill	Sixhill HNB Systems	Modular heating platforms	Customizable

In THSs, tobacco sticks (TEREA Smartcore™) are heated to a temperature of approximately 350 °C using induction technology, resulting in the generation of an aerosol, not smoke, as with traditional CIGs, where the tobacco is completely burned at temperatures of approximately 700 °C–950 °C (Başaran et al., 2019). As a result, the mainstream emissions produced by THSs contain fewer toxic compounds than CIG smoke. Several reports indicated 70%–98% reduction in acrolein, aldehydes, volatile organic compounds, tobacco-specific nitrosamines, carbon monoxide, oxides of nitrogen and sulfur, and heavy metals in the THS emissions (Başaran et al., 2019; Upadhyay et al., 2023). Previously, our group also reported ⁓84% reduction in carcinogenic toxicants in THS emissions compared to those in CIG smoke (Sood et al., 2024). Importantly, the nicotine content in THS emissions and CIG smoke was almost the same, making THSs a viable alternative for individuals addicted to nicotine (Mallock et al., 2018).

The impact of THS emissions on memory and cognition has received very little attention in published literature so far. Thus, it is crucial to carry out research into how THS emissions affect memory and cognition. The aim of the present study was to evaluate the effect of long-term exposure (60 days) to mainstream THS emissions on the memory and cognition of rats. CIG smoke exposure and normal air exposure (control) were used for comparison. Considering long-term exposure and a large number of animals, whole-body exposure instead of nose-only inhalation was followed (Dos Reis et al., 2022). The novel object recognition test (NORT), actophotometer, and forced swim test (FST) were performed to assess memory and cognition. Hematological parameters and body weight were determined for safety. Serum nicotine, cotinine, and brain dopamine were determined for correlation. Furthermore, histopathology of the hippocampus and cortex region of the brain was performed to observe morphological or cellular changes following exposure.

## 2 Materials and methods

### 2.1 Smoking devices

TEREA Smartcore Sticks™ of Philip Morris International (Lausanne, Switzerland), the globally largest selling THS, was selected. The stick consists of a metal heating element coated with stainless steel that heats the tobacco specifically using the IQOS ILUMA induction-heated tobacco device. Comparison was made with commonly available cigarette sticks (CIGs) purchased from the local market. The nicotine per THS and CIG stick was 0.5 mg–4 mg and 4 mg–10 mg, respectively.

### 2.2 Experimental animals

Forty-five adult male albino Wistar rats (150 g–200 g) were housed in a controlled environment at 25 °C ± 2 °C temperature, 57% ± 7% relative humidity, a 12-h light/dark cycle with standard hygienic conditions, free access to water, and an *ad libitum* pelleted diet. All animal experiments were performed as per the guidelines of the Committee for Control and Supervision of Experiments on Animals (CCSEA) with the approved protocol (RRI/IAEC/2024/02) of Rodent Research India, Haryana. The animals were acclimatized for 2 weeks before the experiment. Animals were randomized and divided into three groups (n = 15). Group A consisted of control rats, group B consisted of rats exposed to THS emissions, and group C consisted of rats exposed to cigarette smoke. Animals were sacrificed by cervical dislocation under light anesthesia after the completion of exposure for 60 days. The female research worker who performed the behavioral study and collected the blood and tissue samples was aware of the groups (open-label), but the male research worker who performed the scoring and analyzed the samples was not aware of the same (blinded).

### 2.3 Emission/smoke exposure protocol

**FIGURE 1:**
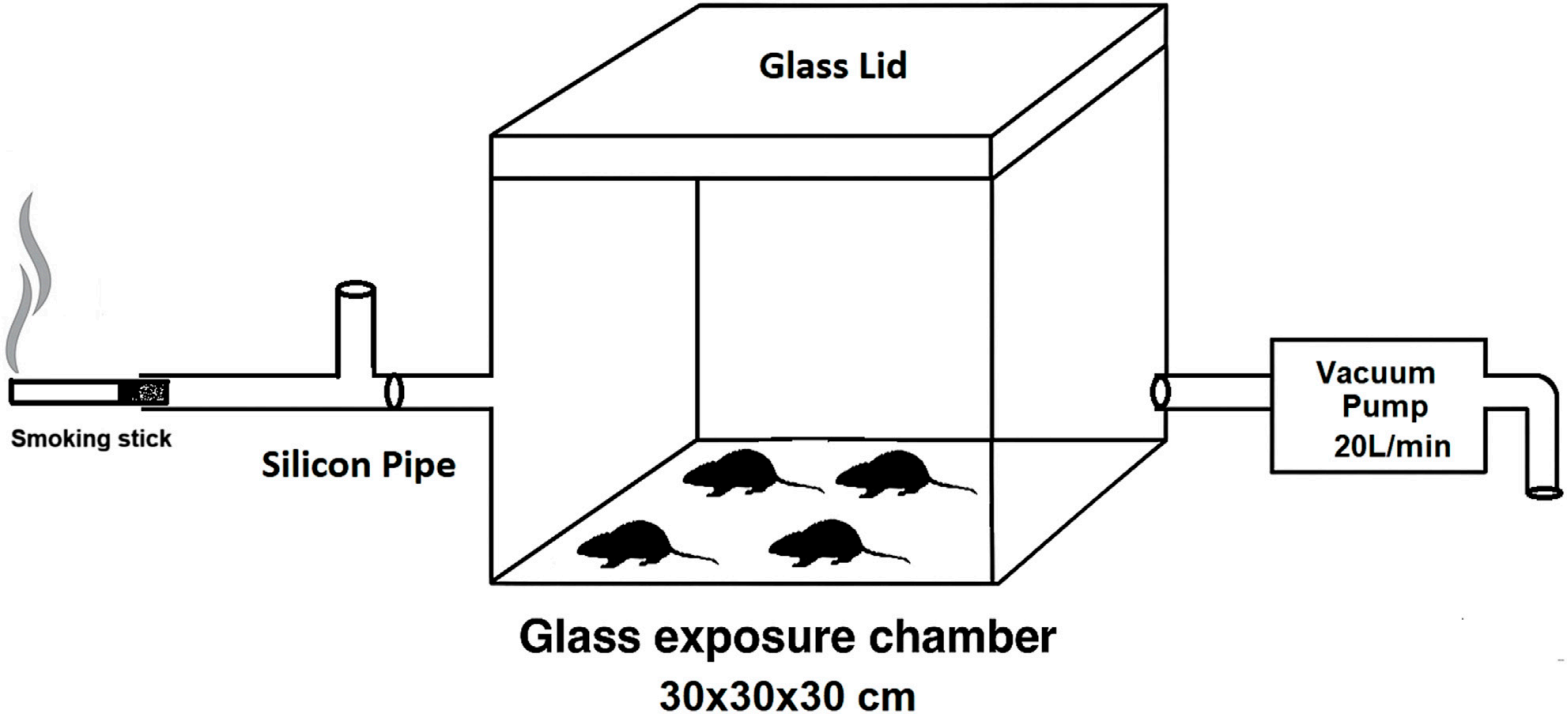
Schematic representation of the whole-body exposure system used for the exposure of rats to mainstream emissions/smoke.

The main elements in our whole-body exposure system include a T-tube, a vacuum pump, and an exposure chamber (Figure 1). Compelled by the vacuum pump, the emissions or smoke moves uni-directionally from the T-tube to the exit valve. The smoking stick burns outside the exposure chamber in a glass T-tube, which allows mixing between air and flames, thus keeping the stick heating/burning. A stick takes ∼4 min to burn completely using a vacuum pump maintained at a flow rate of 20 L/min. The T-tube is connected to the exposure chamber via a silicone tube (5 mm × 40 mm). The exposure chamber is made up of glass (30 cm × 30 cm × 30 cm) and has a glass lid. During experimentation, the lid was sealed with silicone sealant to prevent air leakage from the exposure chamber. The vacuum pump is connected to the exposure chamber via a silicone tube (5 mm × 80 mm) and syringe filter (0.22 µ). The emissions/smoke exits from the other end of the vacuum pump into the water through a silicone tube. Four rats were simultaneously exposed to emissions or smoke, and the exposure was whole-body. Two tobacco sticks per day, one in the morning and one in the evening, were used daily for 60 days. The total exposure time per day was 8 min. The exposure method was modified from the previous report (Dos Reis et al., 2022). All the experiments were conducted in a controlled temperature of 25 °C ± 2 °C and relative humidity of 50% ± 5%, which was maintained by an air conditioner.

### 2.4 Toxicant assessment

Nicotine-free dry particulate matter (NFDPM) was determined by the change in the weight of the filter paper before and after smoking. The total particulate matter (2.5 µm) was determined by Temtop M2000 Air Quality Monitor (Elitech Technology Inc., United States). The total volatile organic compounds were determined using gas chromatography–mass spectrometry (GC-MS, QP-2010 Plus, Shimadzu, Japan). Acrolein in the emission was determined by gas chromatography using a nitrogen-specific detector. Oxidants of nitrogen, sulfur dioxide, and ammonia were detected by an auto-analyzer (EECL, India). Hydrogen sulfide was determined by ion chromatography, and hydrogen cyanide was quantified by spectrophotometry. Lead was detected by the colorimetric dithizone method, whereas arsenic was determined by anodic stripping voltammetry. Toxicants from the mainstream emissions were collected using either a glass fiber filter or a suitable absorption solvent (Sood et al., 2024). The procedures mentioned in the National Institute for Occupational Safety and Health (NIOSH) Manual of Analytical Methods (NMAM) fifth edition were adopted (Ashley, 2015).

### 2.5 Body weight assessment

Changes in the body weight of all three groups of rats were investigated before and after exposure to mainstream emissions/smoke. The percent change in body weight (% Δ BW) was calculated according to the following Equation 1:

$$\%∆\text{BW}=\frac{{\text{BW}}_{1}‐{\text{BW}}_{o}}{{\text{BW}}_{o}}\times 100,$$ (1)

where BW1 is the body weight at a given time point and BW0 is the initial body weight.

### 2.6 Cognitive performance

The long-term impact of emissions/smoke exposure on the behavior and cognitive function was investigated using the NORT, actophotometer, and FST. All the cognitive performance studies were conducted post-exposure.

#### 2.6.1 Novel object recognition test (NORT)

The NORT was used to evaluate recognition memory in rats by observing their ability to distinguish a novel object from a familiar one. The NORT comprised three sessions: habituation, familiarization, and testing. The test was conducted in a black open-field box (Bekci et al., 2024). During the habituation phase, each rat was placed in the empty open field and allowed to explore for 10 min. In the familiarization phase, two identical objects were placed on the left and right sides of the open field. The mouse was placed in the arena with its head facing away from the objects and allowed to explore freely for 10 min. After 24 h, the test session was conducted by replacing one of the identical objects with a novel object. Each rat was then allowed to explore for 10 min in the open field. Throughout all test phases, the objects and the open-field arena were cleaned with 70% v/v alcohol to eliminate olfactory cues. The time spent in exploring both the familiar and novel objects was recorded, and the discrimination index (DI) was calculated using the following Equation 2:

$$DI=\frac{\left(\text{Time}\;\text{devoted}\;\text{to}\;\text{the}\;\text{novel}\;\text{object}\;\left(\text{TN}\right)-\text{Time}\;\text{devoted}\;\text{to}\;\text{the}\;\text{familiar}\;\text{object}\;\left(\text{Tf}\right)\right)}{\left(\text{Time}\;\text{devoted}\;\text{to}\;\text{the}\;\text{novel}\;\text{object}\;\left(\text{TN}\right)+\text{Time}\;\text{devoted}\;\text{to}\;\text{the}\;\text{familiar}\;\text{object}\;\left(\text{Tf}\right)\right)}.$$ (2)

The negative DI value indicates a preference for the familiar object, 0 indicates no preference for either object, and a positive value indicates a preference for the novel object (Bekci et al., 2024).

#### 2.6.2 Forced swim test (FST)

The FST is a behavioral test in small animals used primarily to assess antidepressant efficacy. However, the test is also linked to cognitive performance, particularly in pre-attentive processes and spatial memory. Briefly, rats were dropped one at a time in a plexiglass cylinder (height 50 cm and diameter 30 cm) containing water up to the height of 40 cm at room temperature and forced to swim for 6 min. After a brief spell of vigorous activity, they showed a posture of immobility, characterized by floating motionless in the water and making efforts only to keep the head above water. This immobility reflects a state of depression. After allowing 1 min for acclimatization, each animal was observed for 5 min for immobility. Thus, the immobility time (i.e., the total duration of immobility in a period of 5 min) was recorded for each animal (Becker et al., 2023).

#### 2.6.3 Actophotometer

An actophotometer, primarily used to measure locomotor activity, can provide insights into cognitive performance as changes in movement patterns can reflect alterations in alertness and cognitive processes. An actophotometer (Orchid Scientific, Nashik, India) consists of a digital counter, a photocell, and a light source. Briefly, the activity score for all animals was recorded by placing each animal individually in an actophotometer for 5 min. Comparison was made with the control group, and an increased activity score was taken as an index of CNS stimulation (Bhosale et al., 2011).

### 2.7 Leukocyte count and hemoglobin

Post-exposure, 100 µL of blood was drawn and utilized for measurements of leukocytes and hemoglobin (Hb) using a hematology analyzer (Sysmex America Inc., United States).

### 2.8 Serum nicotine and cotinine levels

The nicotine and cotinine concentration in rat serum was estimated by HPLC (Shimadzu, Japan). The analysis was performed in isocratic conditions using a C18 column (5 µm) at 37 °C using a mixture of 95% (v/v) water containing potassium dihydrogen phosphate (0.272 g) and hexane sulfonic acid (0.184 g), with 5% (v/v) methanol as the mobile phase. The pH of the mobile phase was adjusted to 3.2 with orthophosphoric acid. The flow rate used was 1.0 mL/min, and the wavelength was 256 nm, as per the previous report (Alhusban et al., 2023). Hundred microliters of the serum sample was vortexed with five hundred microliters of acetonitrile for 2 min followed by centrifugation at 15,000 rpm at 4 °C. Thereafter, the supernatant was collected and evaporated under nitrogen and reconstituted in 120 µL of the mobile phase, and then, 100 µL was injected into the separation column for analysis (Alhusban et al., 2023).

### 2.9 Brain dopamine levels

The dopamine level in the rat brain was estimated using the HPLC system (Shimadzu, Japan). The mobile phase consisted of a mixture of 97% (v/v) 0.05 M citrate buffer and 3% (v/v) methanol. The pH of the mobile phase was adjusted to 5.2 with 1N NaOH and 1N HCl. The flow rate used was 1.0 mL/min, and the wavelength was fixed at 256 nm for dopamine, as per the modified method (Tesoro et al., 2022). For sample preparation, 200 mg of brain tissue was placed in 2 mL of the mobile phase (10% w/v tissue homogenate) and homogenized over an ice bed. The homogenized tissue sample was centrifuged at 15,000 rpm and 4 °C for 15 min. The supernatant was taken and filtered using a 0.22-mm syringe filter, and then 20 µL was injected into the HPLC system for analysis.

### 2.10 Hippocampus and cortex histopathology

The change in the morphology of the hippocampus and cortex due to the exposure to THS emissions or CIG smoke was evaluated using histopathological analysis. Briefly, brain tissue was carefully excised and dissected to expose the hippocampus and cerebral cortex. The tissues were separated and fixed in 10% neutral buffered formalin, embedded in paraffin, and horizontally cut into 5-μm-thick sections. The tissues were stained with hematoxylin–eosin. Sections were observed by a trinocular microscope (Evident Scientific, Tokyo, Japan) at ×100 magnification. Ten visual fields were analyzed per tissue section from each animal.

### 2.11 Statistical analysis

The data were expressed as the mean ± SD and analyzed using one-way ANOVA with a *post hoc* Tukey test using GraphPad Prism 8 software. The criterion for statistical significance was p < 0.05.

## 3 Results

### 3.1 Toxicant assessment

THS emissions showed significantly lower toxicant levels than CIG smoke (Table 2). NFDPM was ∼6-fold lower; particulate matter was ∼10-fold lower; and nitrogen/sulfur oxides, ammonia, hydrogen sulfide, hydrogen cyanide, and arsenic were significantly reduced (p < 0.001). Notably, lead traces were detected in CIG smoke but not in THS emissions. Similar observations were reported previously when toxicants in CIG smoke and THS emissions were compared (Sood et al., 2024).

**TABLE 2:** Toxicant levels in THS emissions and CIG smoke.

Toxicant	CIG (mean ± SD)	THS (mean ± SD)
Nicotine-free dry particulate matter (NFDPM) (mg)	13.1 ± 0.8	2.19 ± 0.05*
Particulate matter (ppm)	0.19 ± 0.05	0.02 ± 6.8#
Oxidant of nitrogen (ppm)	143 ± 9.2	16 ± 6.8^#^
Sulfur dioxide (ppm)	1.41 ± 0.23	0.46 ± 0.11^#^
Ammonia (ppm)	49.2 ± 4.8	16.2 ± 3.1^#^
Hydrogen sulfide (ppm)	1.73 ± 0.1	0.06 ± 0.02#
Hydrogen cyanide (ppm)	0.18 ± 0.08	0.06 ± 0.05*
Total volatiles (ppm)	5.23 ± 0.14	3.81 ± 0.12*
Acrolein (ppm)	5.23 ± 0.14	0.81 ± 0.12
Lead (ppb)	0.93 ± 0.11	n.d.
Arsenic (ppm)	0.85 ± 0.07	0.1 ± 0.03#

*p < 0.05, #p < 0.001 vs. CIG.

### 3.2 Body weight assessment

**FIGURE 2:**
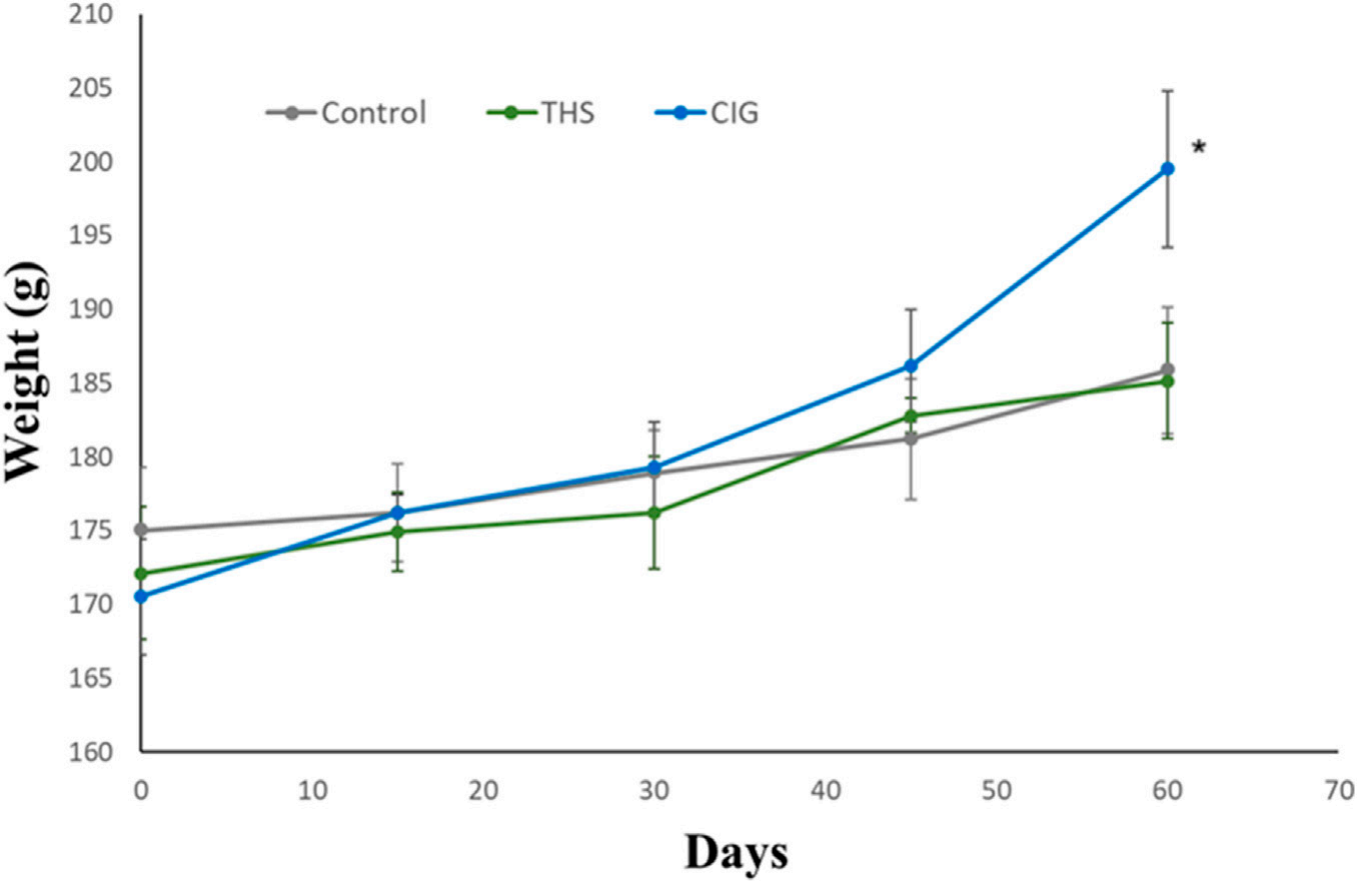
Time-course plot of change in body weight from baseline to day 60 in control and exposed rats. CIG vs. control, p < 0.05; CIG vs. THS, p < 0.05 (at day 60).

The change in the body weight from baseline to post-exposure was determined for all the groups and is presented in Figure 2. An increase in the body weight was observed for all three groups of animals. The increase in the body weight of animals in the THS group was 7.59%, which was not significantly (p > 0.05) different from that in control animals (6.2%). In contrast, animals in the CIG group showed a marked increase in body weight (17.0%), and the values were significantly different (p < 0.05) from those of control and THS animals.

### 3.3 Cognitive performance

#### 3.3.1 NORT

**FIGURE 3:**
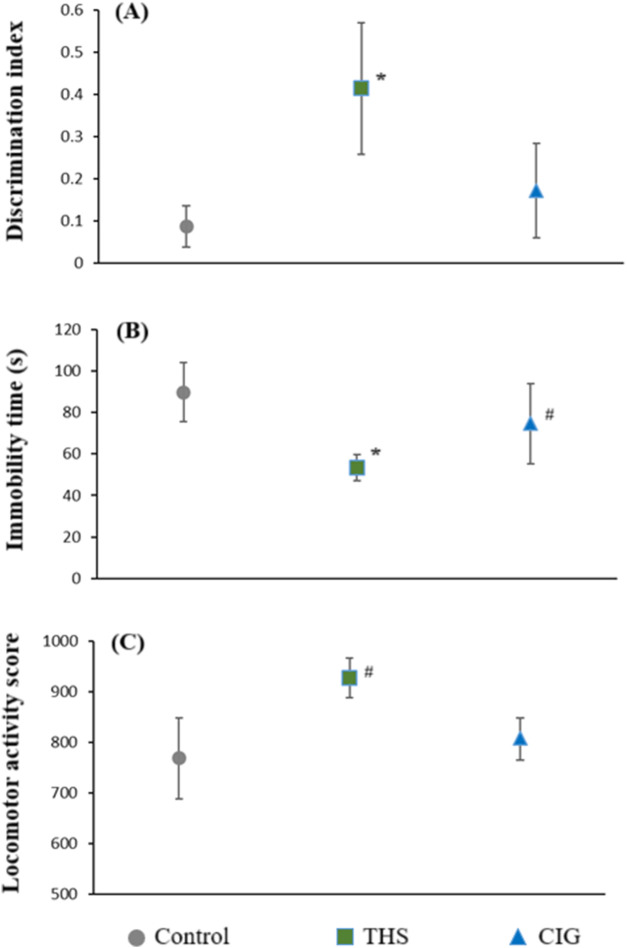
Cognitive performance parameters of the control and exposed rats **(A)** discrimination index (NORT), **(B)** immobility time (FST), and **(C)** locomotor activity (actophotometer). *p < 0.01 vs. control; ^#^p < 0.05 vs. control.

The DI for control, CIG, and THS groups was 0.086 ± 0.049, 0.171 ± 0.112, and 0.414 ± 0.156, respectively (Figure 3A). Our findings revealed that THS emissions, but not CIG smoke, cause a significant increase (p < 0.01) in the DI value compared to that in control animals. This may translate into improvement in the recognition memory of rats following exposure to THS emissions.

#### 3.3.2 FST

The immobility time as determined by the FST for all three groups is shown in Figure 3B. Our results showed that THS emissions and CIG smoke exposure resulted in a decrease in immobility time compared to that in the control (89.8 ± 14.18). As expected, the immobility time was considerably lower after exposure to THS emissions (53.4 ± 6.50, p < 0.01) compared with exposure to CIG smoke (74.6 ± 19.28). A significant decrease in immobility time compared to that in the control group (89.8 ± 14.18) indicates an antidepressant-like effect of THS exposure, suggesting improved coping behavior and reduced behavioral despair.

#### 3.3.3 Actophotometer

Actophotometer scores for all three groups of animals are presented in Figure 3C. The locomotor activity of the control animals was 768 ± 80.42. A significant (p < 0.05) increase in locomotor activity was observed following exposure to THS emissions (927 ± 28.86) compared with exposure to CIG smoke (807 ± 21.67). THS exposure led to a moderate elevation in locomotor activity compared to that in the control, which was indicative of mild CNS arousal, without evidence of hyperactivity or anxiety-like behavior.

### 3.4 Hemoglobin and leukocyte count

**FIGURE 4:**
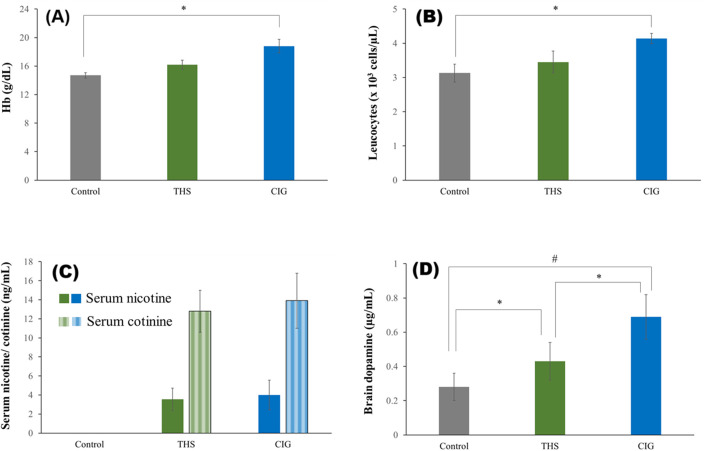
Biochemical levels of the control and exposed rats’ **(A)** Hb, **(B)** leukocyte count, **(C)** serum nicotine and cotinine, and **(D)** brain dopamine. ^*^p < 0.05; ^#^p < 0.001.

The Hb and leukocyte count of the control and exposed rats is shown in Figures 4A,B, respectively. Both Hb and leukocytes were higher in exposed rats than in the control rats. The Hb level of the CIG group rats was 18.8 ± 0.95 g/dL, which was markedly higher than that of the control rats (14.7 ± 0.38 g/dL). The Hb level for THS rats was 16.2 ± 0.63 g/dL, and the difference from the control group was not significant. The leukocyte count of the control group rats was 3.13 ± 0.26 × 10^3^ cells/µL, which increased to 3.45 ± 0.32 × 10^3^ cells/µL for the THS group rats and 4.14 ± 0.15 × 10^3^ cells/µL for the CIG group rats. The difference between the CIG and control rats was significant (p < 0.05).

### 3.5 Serum nicotine and cotinine

The concentration of nicotine and cotinine in the serum of rats exposed to THS emissions and CIG smoke is shown in Figure 4C. As expected, the serum nicotine was higher for the CIG group (3.99 ± 1.56 ng/mL) than for the THS group (3.55 ± 1.17 ng/mL), but the difference was not significant (p > 0.05). As expected, nicotine was not detected in the control rats. Similar results were obtained for serum cotinine levels (Figure 4C).

### 3.6 Brain dopamine

The amount of dopamine in the brains of control rats and rats exposed to THS emissions and CIG smoke is shown in Figure 4D. Expectedly, the brain dopamine level of rats exposed to either THS emission (0.43 ± 0.11 mcg/mL) or smoke (0.69 ± 0.13 mcg/mL) was markedly higher than that of control rats (0.28 ± 0.08 mcg/mL). Interestingly, the brain dopamine level in CIG smoke-exposed rats was significantly higher (p < 0.05) than that in THS emission-exposed rats.

### 3.7 Hippocampus and cortex histopathology

**FIGURE 5:**
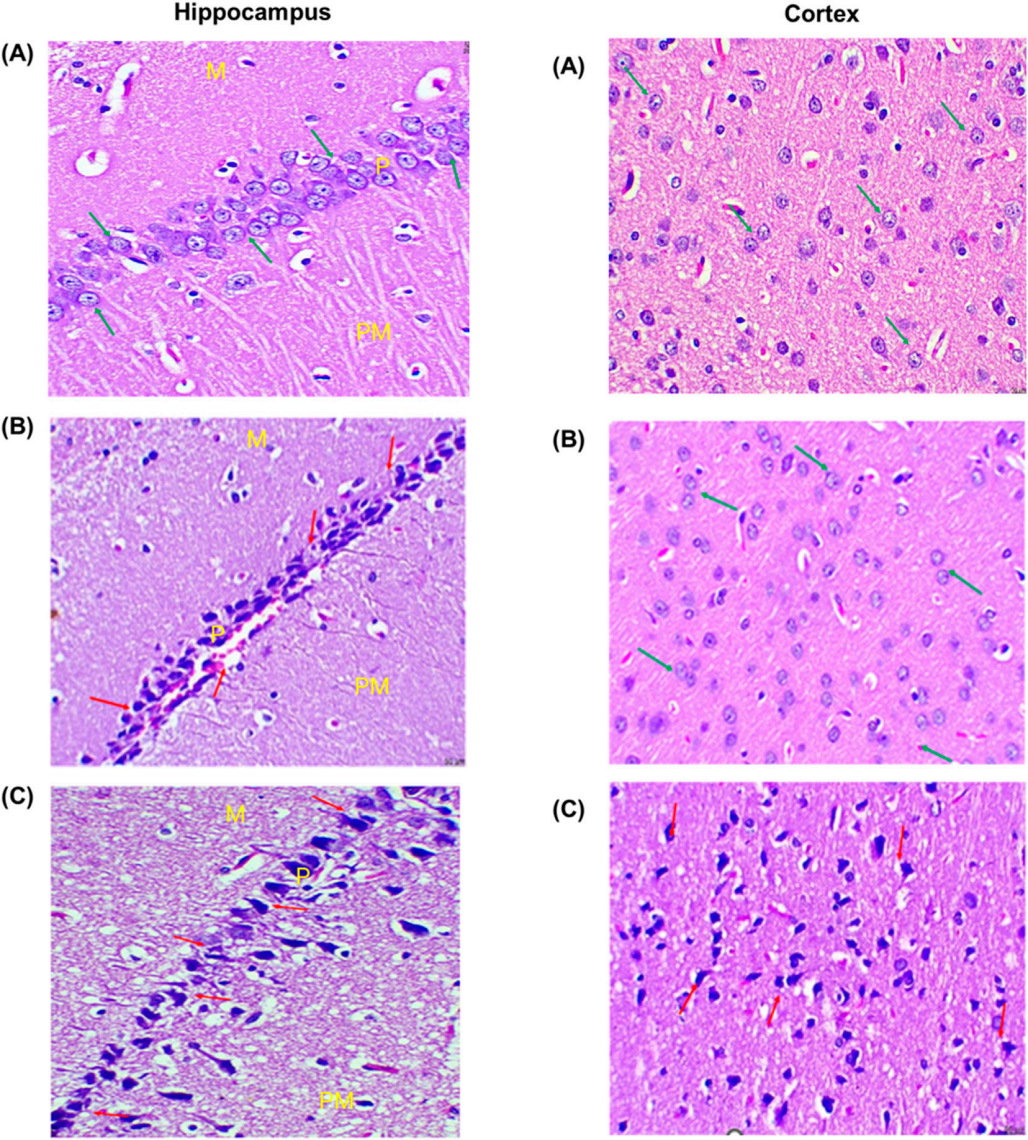
Histopathology images of the hippocampus and cortex region of the brain of the **(A)** control, **(B)** THS, and **(C)** CIG group rats. (H&E, 100x, 50 µm). Hippocampus histopathology images showed molecular (M), pyramidal (P), and polymorphic (Po) layers. The glial cells and capillaries are scattered inside molecular and polymorphic layers. Cortex histopathology showed astrocytes and neurons.

The cellular morphology of the hippocampus and cortex after exposure to THS emissions and CIG smoke was evaluated using histopathology. The changes in cellular morphology of the hippocampus and cortex were observed in exposed rats and compared to those of control rats (Figure 5A). The hippocampus histopathology images of control rats showed organized and structured pyramidal cells and well-distinguished molecular, pyramidal, and polymorphic layers. In the THS group, the disorganization of the pyramidal cell layer was visible with 4% reduction in total cells and 8% reduction in glial cells. No significant changes in capillaries were observed, and the molecular, pyramidal, and polymorphic layers are well distinguished. In contrast, the degeneration of the pyramidal cell layer was visible in the hippocampus of the CIG group rats. An 18% reduction in total cells and a 32% reduction in glial cells were also observed. Furthermore, the presence of vacuolations and neurogliosis was also observed in the CIG group, indicating hippocampus toxicity (Figure 5). Similar observations were visible in the histopathology images of the cortex region, and the animals in the THS group showed a normal cortex with insignificant changes in astrocyte number compared to that in control. On the contrary, the CIG group animals demonstrated astrocyte infiltrations, neuronal degeneration, and the presence of abnormal neurons with flame-shaped processes and faded nuclei with evidence of cortical toxicities (Figure 5).

## 4 Discussion

To assess the potential health risks associated with long-term THS exposure in humans, preclinical investigations are essential. In the present study, we observed that prolonged exposure to THS emissions moderately influenced affective and locomotor behaviors in rats, and further behavioral assessments (such as the NORT) indicated possible improvements in recognition memory.

In this study, using a low-cost, whole-body, glass exposure chamber, we assessed the effects of long-term THS exposure on rats. Owing to the higher prevalence of smoking in males (Erik et al., 2025) and the avoidance of hormonal influence (Hughes, 2019), male rats were used for the study. A prolonged exposure period of 60 days allowed quantitative differences between the groups to emerge. Whole-body exposure instead of nose-only inhalation exposure allows long-term exposure of a large number of animals. We observed tumor development at day 22 in rats exposed to CIG smoke for a long duration, and, therefore, short daily exposure time (⁓8 min/day and two sticks/day) was followed. Unlike the previous studies wherein animals were exposed to second-hand smoke (Raber et al., 2023), we focused on mainstream emission exposure.

Critically, both CIG smoke and THS emissions contain a complex mixture of chemicals, including nicotine. Several preclinical and clinical studies have demonstrated that nicotine exerts cognitive-enhancing effects (Alhowail, 2021; Valentine and Sofuoglu, 2018; Swan and Lessoy-Schlaggar, 2007; Warburton, 1994). Nicotine’s cognitive-enhancing effects are mainly mediated by the stimulation of phosphoinositide 3-kinase/Akt signaling, activation of the brain’s mesolimbic reward circuitry, and release of the brain neurotransmitter dopamine (Swan and Lessoy-Schlaggar, 2007). Analogous to this, following whole-body exposure to THS emissions or CIG smoke for 60 days (2 sticks/day for 8 min), we observed markedly higher serum nicotine and brain dopamine levels compared with those in control rats. The findings were in corroboration with previous results, where long-term CIG or THS exposure showed increased serum nicotine and brain dopamine levels (Herman and Tarran, 2020). Long-term exposure to THS emissions or CIG smoke in rats leads to varying nicotine and cotinine levels, depending on the study’s methodology and duration. The daily short-exposure time and avoidance of second-hand smoke resulted in serum nicotine and cotinine levels that were markedly less than those reported previously (Cano et al., 2020).

Interestingly, despite differences in the claimed nicotine content per stick of THS (0.5 mg–4 mg) and CIG (4 mg–10 mg), we observed similar serum nicotine and cotinine concentration in the exposed rats. Although nicotine and cotinine concentrations were higher in the CIG group rats, the difference was not significant (p > 0.05). The nicotine content in the emissions or smoke generated by tobacco sticks is a highly debated topic, with reports presenting conflicting findings. Whereas some reports suggest that nicotine levels in THS emissions and CIG smoke are comparable (Mallock et al., 2018), others demonstrated lower levels in THS emissions (Goniewicz et al., 2012). It is important to note that the nicotine content in smoking devices is influenced by several factors, including the region of manufacturing, type of tobacco, processing method of tobacco, design of the cigarette, filter used, puff frequency, and depth (Gholap et al., 2020). The nicotine levels can vary distinctly between different CIG brands and between the same brand as well. Goniewicz M.L. et al. (2012) reported high variation in nicotine yields between CIGs of the same brand (RSD, 16%–34%) and between a single CIG of a particular brand (RSD, −65% to +76%) [32]. In another study, nicotine in CIG smoke of several CIG brands was determined, and the results showed markedly high variation, ranging from 0.27 mg to 12 mg nicotine/g tobacco (Carmines and Gillman, 2019).

In contrast to serum nicotine, the brain dopamine level of rats exposed to CIG smoke was significantly higher than that in rats exposed to THS emissions. Dopamine is a key neurotransmitter involved in regulating mood, motivation, and cognitive processes such as attention and memory formation. Elevated dopamine levels have been associated with enhanced cognitive performance and learning capacity (Ashby et al., 2024). To comprehensively evaluate the behavioral impact of THS and CIG exposure, we conducted a series of validated behavioral assays, including the NORT for cognitive assessment, the FST for affective behavior, and the actophotometer test for locomotor activity to distinguish cognitive outcomes from general alterations in mobility or affective state.

The NORT was used to assess memory for object identity in rats (Bekci et al., 2024). The time spent in exploring both the familiar and novel objects was recorded, and the DI was calculated.

We observed a significantly higher discrimination index for THS emission-exposed rats than for CIG smoke-exposed and control rats. This means that THS emission-exposed rats spent significantly more time with the novel object than with the familiar object. Although CIG smoke-exposed rats also spent more time with the novel object, the difference did not reach statistical significance compared to the control. The DI value of the control group is notably low but positive. This observation was observed predominantly because one of the animals in the control group showed a large negative value, shifting the mean toward 0.

Consistent with the behavioral outcomes, THS exposure resulted in a significant reduction in immobility time in the FST compared to both CIG smoke-exposed and control rats. Although decreased immobility time is traditionally interpreted as an indicator of antidepressant-like or stress-coping behavior (Bhosale et al., 2011), earlier studies have also discussed potential overlaps between affective and cognitive processes in such paradigms (West, 1990). However, in accordance with the current understanding, the observed reduction in immobility is best interpreted as reflecting affective behavioral modulation rather than direct enhancement of learning or memory.

To obviate the possibility that improvements observed in the NORT and FST were confounded by altered locomotor activity, we conducted an actophotometer test prior to behavioral assessments. An increased activity score was presumed as an index of CNS stimulation (Becker et al., 2023). It is important to note that the THS-exposed group exhibited a statistically significant increase in locomotor activity compared with both the control and CIG groups (p < 0.01 and p < 0.05, respectively). Although this elevation was interpreted as indicative of mild CNS arousal, it cannot be entirely excluded that the enhanced locomotion may have contributed to the apparent improvements in exploratory and mobility-dependent behavioral tasks such as the NORT and FST. Therefore, the observed cognitive and antidepressant-like effects should be interpreted with caution, acknowledging the possibility of partial locomotor influence on behavioral performance.

Interestingly, despite exhibiting lower brain dopamine levels, THS-exposed rats demonstrated enhanced affective and exploratory behaviors compared with CIG smoke-exposed rats. Although dopamine is well recognized for its role in the reward and addiction mechanisms (Ashby et al., 2024), the present findings suggest that THS exposure may induce less dopaminergic reinforcement potential than CIG, indicating comparatively lower addictive liability rather than direct cognitive enhancement.

Well-documented evidence suggested that CIG smoke and THS emissions, in addition to nicotine, contain numerous chemicals, many of which are toxic. Oxidants of nitrogen, sulfur dioxide, ammonia, hydrogen cyanide, acrolein, particulate matter, and heavy metals present in the smoke/emissions have been linked to neurotoxicity and cognitive decline (Sood et al., 2024). In this study, THS emissions showed significantly lower amounts of toxicants (p < 0.001), including approximately 5-fold lower tar and 2-fold lower particulate matter (2.5 µm) than CIG smoke. The results were consistent with the results of our previous *in vitro* studies (Sood et al., 2024) and support the findings of the study performed by Liu et al. (2024). The presence of higher neurotoxicants and NFDPM in CIG smoke might explain the lower discrimination index (NORT) and less decrease in immobility time (FST) in CIG rats than in THS rats despite higher brain dopamine. The present findings support a previous study where CIG smoke has been shown to trigger oxidative stress, inflammatory response, and damage neurons, resulting in cognitive impairment (Campos et al., 2016; Conti et al., 2019). Toxicants present in CIG smoke are pro-oxidants and oxidants that produce free radicals and enhance oxidative stress *in vivo*. Brain enriched with non-heme iron and polyunsaturated fatty acids is extremely vulnerable to free radicals, and the resulting oxidative stress activates inflammatory mediators and neuronal damage.

In line with this, our histopathology data demonstrated atrophic and apoptotic changes, cellular damage, pyknosis, and toxicity in the hippocampus of CIG smoke-exposed rats. In addition, pronounced cellular disintegration was visible in the cortex region. Notably, the changes were mild in THS emission-exposed rats, and hippocampus and cortex toxicity was absent. As discussed, this could be due to lower toxicants in THS emissions than in CIG smoke. Similar results were published previously (Coggins et al., 1989).

In agreement with this, we also observed a marked rise in the body weight of CIG group rats. The weight increase was significant compared with that in the THS and control rats. Unlike previous reports, where increased serum nicotine causes reduced weight gain in animals chronically exposed to CIG smoke (Wager-Srdar et al., 1984), we observed weight gain in the rats exposed to CIG smoke. The nicotine levels between the CIG and THS groups of our study were not significantly different, and, therefore, it is hypothesized that the presence of toxicants in smoke might be responsible for altered metabolism and weight gain in the CIG group animals.

The theory of inflammatory or immune response generated by CIG smoke exposure was supported by significantly increased leukocyte counts observed in smoke-exposed rats (Koh, 2023). Although the leukocyte count of THS emission-exposed rats was higher than that of control rats, the difference was not significant (p > 0.05). High Hb was observed in exposed rats, and the increase was greater in the CIG group rats. This may be attributed to the presence of CO in smoke/emission, which can diffuse rapidly and bind to Hb, leading to tissue hypoxia because of the formation of carboxyhemoglobin (Malenica et al., 2017). In order to maintain blood homeostasis, the rise in Hb may have occurred. As the level of CO is higher in smoke than in emissions, the rise in Hb was higher in the CIG group rats.

The present study has several limitations. First, the study was conducted only on male rats owing the higher prevalence of smoking in males and the avoidance of hormonal influence. Second, whole-body exposure instead of nose-only inhalation exposure was followed. Whole-body exposure allows long-term exposure of a large number of animals. Furthermore, we observed tumor development in rats exposed to long daily smoke exposure, and, therefore, short daily exposure time (⁓8 min/day) was followed. Finally, we analyzed a few relevant toxicants in mainstream emissions/smoke. Our previous *in vitro* studies compared the concentration of 49 toxicants in mainstream emissions and smoke.

## 5 Conclusion

This study is among the first to comparatively evaluate the long-term effects of THS emissions and CIG smoke exposure on behavioral and neurochemical outcomes in rats. The findings indicate that THS emissions, characterized by significantly lower levels of toxicants and particulate matter, elicited milder neurobehavioral alterations and reduced dopaminergic reinforcement potential relative to CIG smoke, suggesting comparatively lower addictive liability. Although THS exposure influenced affective and exploratory behaviors, no direct evidence of enhanced memory or cognitive performance was established.

The observed effects of THS on the CNS may involve noninflammatory or neurotransmitter-mediated mechanisms. However, further mechanistic and behavioral studies are required to comprehensively delineate the neurobiological pathways and long-term safety implications of THS exposure on the CNS.

## Data Availability

The original contributions presented in the study are included in the article/Supplementary Material; further inquiries can be directed to the corresponding author.
